# Hematochezia in a Child With Heiner Syndrome

**DOI:** 10.3389/fped.2019.00551

**Published:** 2020-01-28

**Authors:** Xiang-Yu Liu, Xi-Ru Huang, Jie-Wei Zhang, Yong-Mei Xiao, Ting Zhang

**Affiliations:** ^1^Department of Gastroenterology, Hepatology, and Nutrition, Shanghai Children's Hospital, Shanghai Jiao Tong University, Shanghai, China; ^2^Shanghai Jiao Tong University Affiliated Sixth People's Hospital, Shanghai, China; ^3^Ruili Maternal and Child Care Service Center, Ruili, China

**Keywords:** Heiner syndrome, hematochezia, cow's milk, food hypersensitivity disease, pulmonary infiltrates

## Abstract

Heiner syndrome (HS) is a food hypersensitivity disease that is mostly caused by cow's milk. The main features may include chronic or recurrent respiratory syndromes, pulmonary infiltrates on radiography, and even pulmonary hemosiderosis. However, gastrointestinal symptoms are rare in HS, which can lead to a misdiagnosis when the chief complaint is about the gastrointestinal system. Here, we report a case of HS complicated by severe hematochezia.

## Introduction

Since the discovery of a pulmonary syndrome induced by cow's milk, only a few cases have been published. Heiner syndrome (HS) is caused by cow's milk, first described by Heiner et al. (1) in seven infants in 1962. HS can be resolved by removing cow's milk from the diet, and its manifestations include chronic respiratory symptoms, infiltrates on chest roentgenograph, failure to thrive, pulmonary hemosiderosis, and even iron deficiency anemia (2, 3). However, few gastrointestinal manifestations associated with HS have been reported in the available literature so far (4). Here, we report a case, whose chief presenting symptom was severe hematochezia instead of respiratory symptoms, which is rarely reported in cases of HS.

## Case Report

A 4-month-old girl was referred to our hospital with continuous hematochezia since 10 days after birth, with hematochezia showing signs of aggravation 4 days before being examined. She was born full term (G1P1) without complications and was fed with the mother's milk and cow's milk. Physical examination showed a height of 59 cm, a weight of 4.25 kg, a fever at 38.1°C, and respiratory rates 70 times/min with rough breath sounds. The skin was pale and showed no rashes. Tonsils were not hypertrophic nor inflamed.

**Figure 1:**
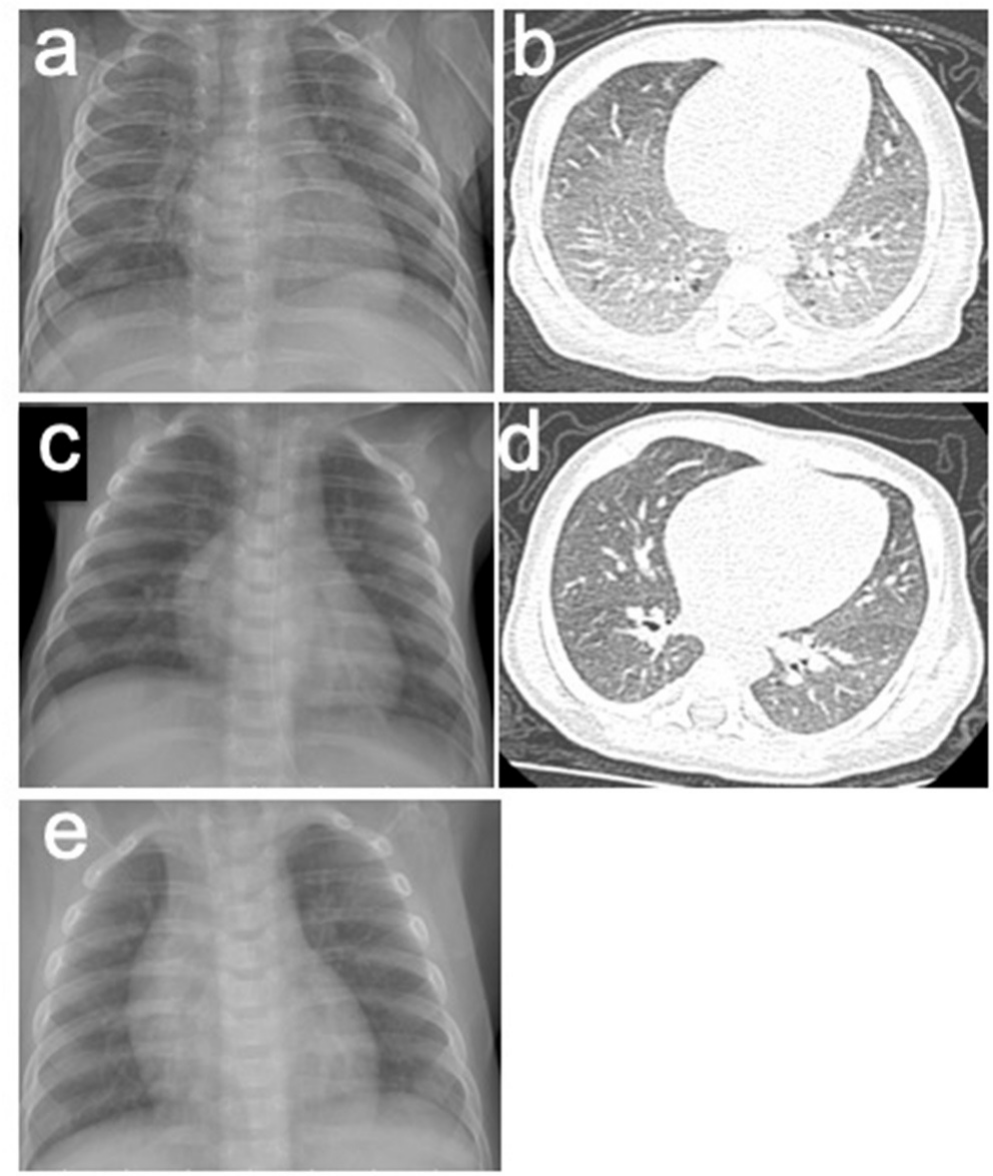
**(a,b)** X-rays and CT scans of the patient before the treatment of HS. **(a)** Chest roentgenogram showed bilateral infiltrates and opacities in different lung lobes. **(b)** Chest CT scan showed interstitial lung disease. **(c,d)** X-rays and CT scans of the patient after the treatment of HS. **(c)** Chest roentgenogram showed that pulmonary infiltrates cleared. **(d)** Chest CT scan was normal. **(e)** Chest roentgenograms in regular follow-up visit showed no infiltrates and opacities.

Laboratory studies showed the following: white blood cells (WBC), 16.31 (10^9^ cells/L) (normal, 8–12); neutrophil, 31%, lymphocytes, 51%, and monocytes, 13% (normal: 3–8); eosinophils, 6% (normal: 1–5); C-reactive protein, 16 mg/L (normal: 0–5), red blood cells, 2.44 (10^12^ cells/L) (normal: 4–5.5); and hemoglobin, 70 g/L (normal: 110–160). Immunoglobulin E (IgE) antibodies to cow's milk is negative, and serum total IgE level was 35.5 IU/mL (normal: < 15). In addition, fecal occult blood was positive. The concentration of iron element in serum was 4.36 mmol/L (normal: 7.52–11.82). The PO_2_ in peripheral blood was 59 mmHg (<60), and PCO_2_ was normal, which indicated type I respiratory failure. Chest roentgenograms showed bilateral infiltrates and opacities in different lung lobes (Figure 1a). The CT scan showed interstitial lung disease (Figure 1b), and the oxyhemoglobin saturation (SpO_2_) could not be maintained without oxygen intake (the lowest SpO_2_ was 80%).

**Figure 2:**
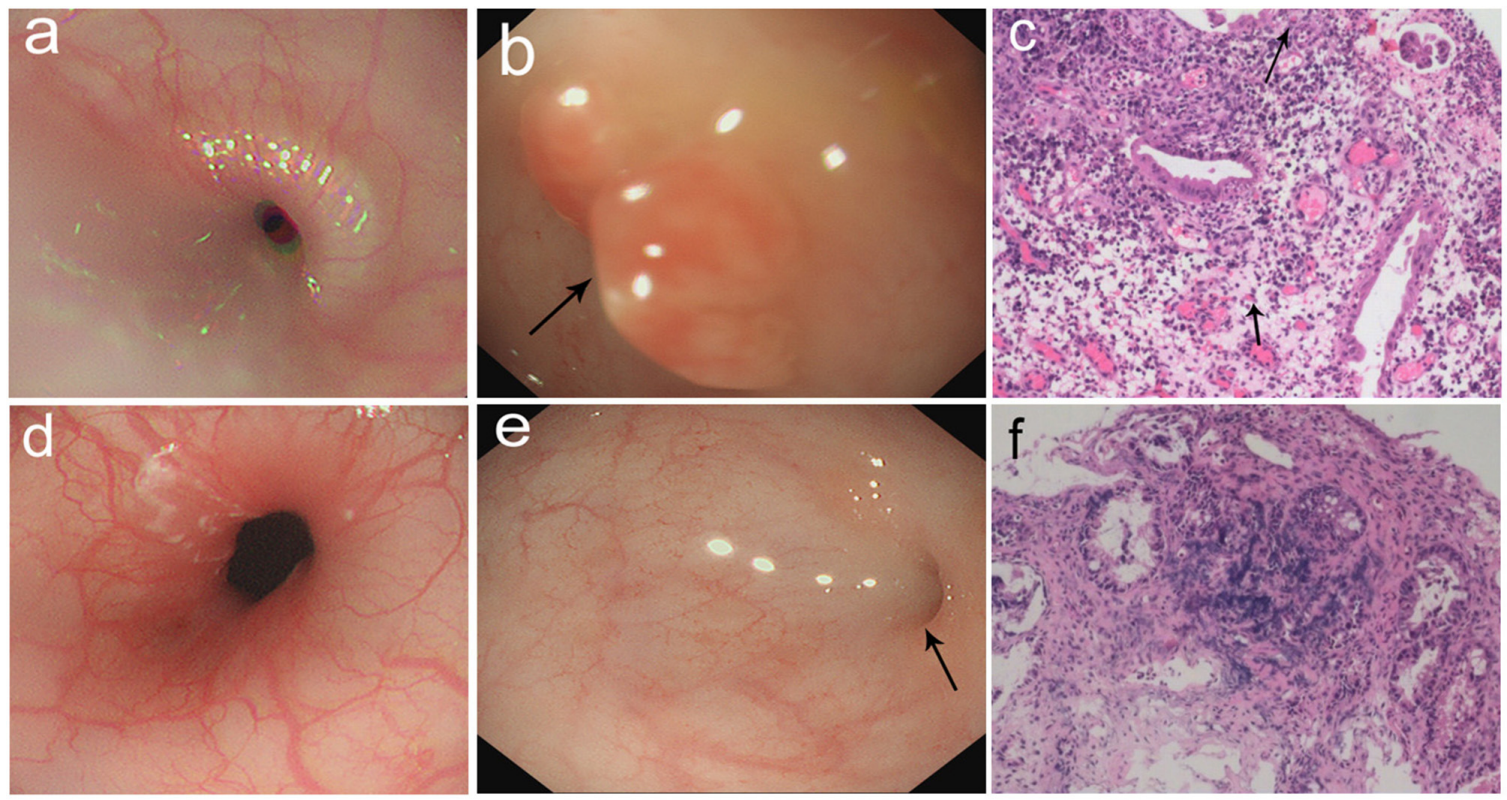
**(a–c)** Gastrointestinal endoscopic pictures and the pathological sections. **(a)** Gastroscopic pictures showed chronic superficial gastritis. **(b)** Enteroscopic pictures showed a hyperplasia in descending colon (arrow: hyperplasia). **(c)** The section of hyperplasia showed granulation tissue infiltrated mostly by acute and chronic inflammatory cells (arrow: eosinophils). (100×) **(d–f)** Gastrointestinal endoscopic pictures and the pathological sections in follow-up visit. **(d)** Gastroscopic pictures showed normal results. **(e)** Enteroscopic pictures showed inflammatory hyperplasia (arrow: hyperplasia). **(f)** The section of hyperplasia showed tissue infiltrated by inflammatory cells. (100×).

The patient was suspicious of HS due to the chronic pulmonary syndrome and the possible history of cow's milk allergy. A low dose of methylprednisolone (1 mg/kg) and montelukast sodium were prescribed. Milk was eliminated from the diet, and amino acid formula was prescribed. Shortly after this, dyspnea significantly improved, and eosinophils decreased to 1%. Chest roentgenograms showed that pulmonary infiltrates cleared within 5 days (Figure 1c), and the CT scan was normal (Figure 1d). In addition, hematochezia was relieved, and hemoglobin was increased to 80 g/L. The diagnosis was further confirmed based on the positive response to the treatment and the elimination of milk. She was subjected to gastrointestinal endoscope, revealing chronic superficial gastritis (Figure 2a) and hyperplasia in the descending colon (Figure 2b). We did an endoscopic protractor biopsy on the hyperplasia. The examination showed granulation tissue infiltrated by acute and chronic inflammatory cells, including some eosinophils (at most three per high-power field) (Figure 2c). In addition, the iron-laden macrophages test in sputum or fasting gastric fluid was negative. She was discharged with low-dose methylprednisolone (4 mg qd), with the dose of methylprednisolone decreased every 2 weeks (4 mg qd→ 4 mg/2 mg qd→ 2 mg qd→ 2 mg qod).

After 2 months, she came back for a regular follow-up. At this time, physical examination showed weight of 6.9 kg, no fever, and respiratory rates 26 times/min. Lungs were clear to auscultation. Skin was not pale and showed no rashes. Tonsils were not hypertrophic and not inflamed. SpO_2_ is more than 98%. Laboratory studies showed that WBC, C-reactive protein, red blood cells, and hemoglobin are all in a normal range. In addition, hematochezia disappeared, and fecal occult blood was negative. Chest roentgenograms showed neither infiltrates nor opacities (Figure 1e). Besides this, the gastroscope showed normal results (Figure 2d), and enteroscope showed inflammatory hyperplasia (Figure 2e). The light microscopic examination of the biopsy showed tissue infiltration by inflammatory cells (Figure 2f). In addition, the enteroscope examination and biopsy also showed that there was lymphonodular hyperplasia, which is uncommon in HS. It may be induced by the delayed-type food hypersensitivity (5).

## Discussion

Our patient mimicked a clinical picture of bacillary dysentery or inflammatory bowel disease with symptoms including hematochezia, diarrhea, and elevated WBC and C-reactive protein. The stool routine test had no significance besides being positive for fecal occult blood, and stool bacterial culture showed no pathogens such as *Shigella, Salmonella, Vibrio cholerae*, or pathogenic *Escherichia coli*. Results of tests and the pulmonary symptoms were not concordant with infection for this age group. In addition, the gastrointestinal endoscopic pictures showed non-specific inflammation, and histological sections showed infiltration of acute and chronic inflammatory cells, and some eosinophils, which indicated allergic proctocolitis. However, only allergic proctocolitis itself cannot account for the pulmonary symptoms in this patient.

The hematochezia after cow's milk diet, the increase in eosinophils, and the anemia indicated an allergy to cow's milk [cow's milk protein allergy (CMPA)]. In addition, the dramatic radiological and clinical improvements of pulmonary and gastrointestinal symptoms after the elimination of cow's milk and the treatment of methylprednisolone supported HS. It is noticeable that this patient had the chief complaint as severe hematochezia instead of respiratory symptoms, which is rarely reported in cases of HS. We also found that there was hyperplasia in intestinal mucosa under enteroscope and lymphonodular hyperplasia in biopsy, which are also rare in HS. It was reported that some people react to some food as though they were pathogens, which can cause hyperplasia with chronic inflammation in the mucosa (6). About the possible mechanisms, mast cells may be stimulated by the antigen–antibody complex and produce cytokines like interleukin-3 and interleukin-5, which promote allergy into a last phase (6, 7), and uncontrolled activated T-cell-mediated process also plays a significant role in hyperplasia and mucosal lesion (6, 8, 9). Lymphonodular hyperplasia can also be a potential source of rectal bleeding of infants (6, 10), and infants with severe rectal bleeding should be undergo an enteroscope examination and biopsy. If lymphonodular hyperplasia is found, delayed-type food allergy should be considered.

The precise mechanism which is responsible for HS is still poorly understood (11). It now seems that HS contains both IgE and non-IgE-mediated allergic responses (12). IgE-mediated reactions can develop when cow's milk-specific IgE antibodies residing on mast cells or basophils come into contact with and bind to the circulating food allergens, such as milk proteins, and then activate immune cells to release allergy mediators such as histamine and cytokines (13). IgE-mediated reactions are characterized by a rapid onset, which usually involves the formation of rashes, urticaria, and oral allergy symptoms. However, in non-IgE-mediated food allergic reactions, multiple inflammatory cells, such as macrophages or lymphocytes, play a significant role in the process (14–16). In addition, lymphonodular hyperplasia, regarded as a histological characteristic of mucosal immune response in the process, may also happen (17–19). In non-IgE-mediated food allergy, disorders in the body become evident after hours or days and predominantly manifest in the gastrointestinal tract, as opposed to the skin. Non-IgE-mediated gastrointestinal allergic disorders contain many diseases, such as enteropathy, enterocolitis, and proctocolitis. The combination of IgE and non-IgE-mediated allergic reactions have been established in the pathogenesis of some diseases, such as eosinophilic esophagitis and gastroenteritis. The exact roles of IgE and non-IgE-mediated allergies during the process of HS needs further investigation as well as specific confirmatory tests aimed to diagnose HS beyond clinical symptoms (3).

## Conclusion

To summarize, HS should be worth considering in any young child who has an unexplained chronic pulmonary infiltrate, respiratory failure, and an evidence of CMPA, even though the chief complaint of the patient is hematochezia and not focused on the respiratory system. This allergy may affect many systems, so extending the focus beyond the chief complaint is imperative. The diagnosis would be supported by dramatic radiological and clinical improvements after the elimination of cow's milk and the treatment of methylprednisolone. Although HS is rare in the pediatric population, this syndrome should be suspected in those who have pediatric pulmonary syndromes and CMPA. Therefore, insufficient awareness about HS is probably a major reason for its misdiagnosis.

## Data Availability Statement

All datasets generated for this study are included in the article/supplementary material.

## Ethics Statement

This study was reviewed and approved by the Ethical Review Commitment of Shanghai Children's Hospital.

## Author Contributions

X-YL drafted the manuscript. TZ revised the manuscript. X-YL, X-RH, J-WZ, and Y-MX collected data and pictures. All authors read and approved the final manuscript.

### Conflict of Interest

The authors declare that the research was conducted in the absence of any commercial or financial relationships that could be construed as a potential conflict of interest.
